# Short-Term Meditation Training Fosters Mindfulness and Emotion Regulation: A Pilot Study

**DOI:** 10.3389/fpsyg.2020.558803

**Published:** 2020-10-26

**Authors:** Teresa Fazia, Francesco Bubbico, Ioannis Iliakis, Gerardo Salvato, Giovanni Berzuini, Salvatore Bruno, Luisa Bernardinelli

**Affiliations:** ^1^Department of Brain and Behavioral Sciences, University of Pavia, Pavia, Italy; ^2^Cognitive Neuropsychology Centre, ASST “Grande Ospedale Metropolitano” Niguarda, Milan, Italy; ^3^Istituto di Psicosintesi, Milan, Italy

**Keywords:** short-meditation training, DSES, CORE-OM, ERQ, FFMQ, mindfulness, general population

## Abstract

The practice of meditation has been historically linked to beneficial effects, not only in terms of spirituality but also in terms of well-being, general improvement of psychophysiological conditions and quality of life. The present study aims to assess the beneficial effects of a short-term intervention (a combination of 12 practical 1-h sessions of meditation, called *Integral Meditation*, and lectures on neuroscience of meditation) on psychological indicators of well-being in subjects from the general population. We used a one-group pretest-posttest quasi-experimental design, in which all participants (*n* = 41, 17 men and 24 women, with a mean age of 41.1 years) underwent the same intervention. Out of these, 24 had already experienced meditation practice, but only 12 in a continuative way. Effects were assessed by the standardized Italian version of three self-report questionnaires: Core Outcome in Routine Evaluation-Outcome Measure (CORE-OM), Five-Facet Mindfulness Questionnaire (FFMQ), and Emotion Regulation Questionnaire (ERQ). The questionnaires were filled in at baseline and immediately after the last meditation session. Linear mixed effect models were used to evaluate pre-post treatment changes on each outcome. Participants showed a general, close to a statistically significant threshold, improvement in the total score of CORE-OM and its different domains. The total score of FFMQ (β = 0.154, *p* = 0.012) indicates a statistically significant increase in the level of mindfulness as well as in the domains *acting with awareness* (β = 0.212, *p* = 0.024), and *non-judging of inner experiences* (β = 0.384, *p* < 0.0001). Lastly, we observed a statistically significant improvement in the *cognitive reappraisal* ERQ domain (β = 0.541, *p* = 0.0003). Despite some limitations (i.e., small sample size, lack of a randomised control group and sole use of “soft” measurements, such as self-report questionnaires), this study offers promising results regarding the within-subject effectiveness of our intervention that includes a meditation practice on psychological indicators, thus providing interesting preliminary results.

## Introduction

Meditation is an ancient practice which has its roots in India about 2,500 years ago and it has been perpetuated until today in various form and through different traditions. Only in the last few decades, meditation has gained consideration in the academic world, given the arising of scientific evidences regarding its beneficial effects on psychological, neurological, endocrine and immune variables, as well as positive influence on well-being and a broad range of biological processes (Paul-Labrador et al., 2006; Rubik, 2011; Bai et al., 2015; Aherne et al., 2016; Chiesa et al., 2017; Wood et al., 2017). Thanks to this evidence, nowdays, meditation practice is frequently integrated in contemporary psychotherapy treatments and used to enhance well-being in several medical conditions and to improve quality of life.

In particular, the practice of meditation has been incorporated in a big family of interventions called mindfulness-based programs (MBPs). A great contribution to the spread of MBPs was given by programs developed for specific populations or contexts, such Mindfulness-Based Stress Reduction (MBSR), and Mindfulness-Based Cognitive Therapy (MBCT). MBSR was developed for people with chronic health problems or suffering from the mounting demands associated with psychological and emotional stress (Kabat-Zinn, 1990, 2006; Kabat-Zinn and Hanh, 2009); MBCT is an approach to psychotherapy that was originally created as a relapse-prevention treatment for individuals with major depressive disorder (Segal et al., 2002; Kabat-Zinn and Hanh, 2009).

Since the MBPs have become so popular, some fundamental questions have been addresses among the researchers, especially on how to define MBP, since nowadays the term mindfulness refers to a multitude of meaning and practices. As discussed in Crane et al. (2017) using a metaphor, authors compare textile manufacture to the “fabric” of MBPs and they called “warp” the essential, constant and integral threads that define an MBP regardless of population or context, while they called “weft” those specific features that characterized each adapted MBP to be a better fit for a particular population or context.

Taken the “warp” elements as references point, we developed a program called *Integral Meditation*, that we integrated in our intervention with lectures about the neuroscience of meditation. Our *Integral Meditation* can be thus considered as MBP with its specific “weft” elements. The goal of our *Integral Meditation* is to give to the person means to maintain emotional balance, to develop the ability to face different relational situations, and skills to deal constructively with stressful and existential situations.

Our intervention was intended to target subjects from the general population (i.e., naive meditators with no important clinical conditions) and the “weft” features of our meditation program were tailored accordingly.

The choice to focus on a general population was motivated by the observation that even though the clinical aspects of meditation are obviously more attractive, a part of the academic world have started to investigate the effects of meditation on non-clinical population with notable and promising results.

Literature focusing on non-clinical population is still scarce than that on clinical setting (Geary and Rosenthal, 2011; Tanay et al., 2012; De Vibe et al., 2013; Khoury et al., 2015); so, our pilot study aimed at contributing filling this gap. Despite not having serious symptoms, sub-samples among the general population can experience stress, anxiety or emotional dysfunction at subthreshold clinical level, with a risk of developing more serious conditions in the future. For example, work-related stress is associated with depression (Tennant, 2001), insomnia (Yang et al., 2018) and cognitive decline (Giorgi et al., 2020) while at the same time it imposes a considerable financial burden on societies (Hassard et al., 2018). Indeed, compelling evidences have shown that meditation brings good outcomes, both in long and short term, to the non-clinical population too by positively affecting brain structure and function (Cahn and Polich, 2006; Tang et al., 2009, 2010; Luders et al., 2011; Davidson and McEwen, 2012), and improving executive functions and cognitive processes (Jha et al., 2007; Moore and Malinowski, 2009; MacLean et al., 2010; Chiesa et al., 2011). Meditation can also reduce the negative dimensions of psychological distress by alleviating anxiety, depression, and pain, relieving stress and improving the mental health component of health-related quality of life (Goyal et al., 2014; Basso et al., 2019). Cultivating a more mindful way of being is associated with a decrease in emotional distress and an increase of positive states of mind (Greeson, 2009). Furthermore, mindfulness meditation practice has been proved effective in reducing ruminative thinking both in healthy subjects other than depressed individuals (Jain et al., 2007). In fact, the state mindfulness stimulated during and immediately after meditation can decrease both stress and anxiety. With repetitive practice, state mindfulness leads to increases in trait mindfulness, and higher levels of trait mindfulness have the same impact of state mindfulness but in a long term period (Bamber and Kraenzle Schneider, 2016).

Anyway, meditation is arguably a practice intended to be trained with motivation and to be perpetuated over time in daily routine to bring solid benefits. Brandmeyer and Delorme (2013) state that despite the increased accessibility and appeal of meditation training, it is still a challenge for many individuals in Western societies to maintain consistent practice. Increasing the motivation toward meditation, such as providing the practitioners scientific reliable information about the effects of meditation, could lead to a higher engagement during the meditation interventions and to an increased possibility that the participants will maintain their interest in attending the intervention sessions and hopefully in maintaining this healthy habit in the future.

Here we report the results of our study aimed at evaluating the within-subject effect of our intervention including a meditation practice interposed to neuroscience of meditation lectures, in a big group on psychological indicators, measured thought self-report questionnaires, and at exploring our *a priori* hypothesis of the existence of a beneficial effect of our intervention on individuals from the general population on such indicators.

## Materials and Methods

### Study Design

We used a one-group pretest-posttest quasi-experimental design, in which all the participants received the intervention (i.e. brief *Integral Meditation* training and lectures on Neuroscience of Meditation) to evaluate its within-subject effect (i.e., before the start of the training vs after the completion of the training) on psychological indicators.

The effects of the proposed intervention were assessed by collecting and scoring the standardized Italian version of three self-report questionnaires (i.e., CORE-OM, ERQ, and FFMQ). All the questionnaires were measured at baseline (*t*_0_) and immediately after the last meditation session (*t*_1_). We also collected and scored the standardized Italian version of the Daily Spirituality Scale (DSES) that was measured once at *t*_0_ and whose score, obtained for each subject, was used as covariate in the statistical models, to take into account the potential effect of spirituality on the outcome. As background variables, we collected age, sex, previous experience of meditation practice and education.

### Participants

Forty-one participants (17 men and 24 female) were recruited from the course “Neuroscience of meditation” that took place at the University of Pavia, Italy, from 23 March to 11 June 2017. The participation to the course was voluntary, and despite it being accessible to everybody, it primarily attracted people with a high level of education and interest in theoretical aspects of meditation. As regard the exclusion criteria, the subjects should not currently experience severe periods of anxiety or depression, severe mental illness (e.g., hypomania or psychotic episode), recent bereavement or major loss, or any other serious mental or physical health problem that would affect their ability to engage with the course. No subjects of the 41 who enrolled for the study were excluded based on the above criteria. For each enrolled subject, data on age, sex, education and previous experiences of meditation were collected. Each subject has to attend at least 75% of intervention sessions to be retained in the study and has to fill in all the questionnaires both at pre and post intervention. All the enrolled subjects have been retained in the study. For each subject, data on age, sex, education, and previous experiences of meditation were also collected.

### Intervention

Our intervention was structured into five lectures on the neuroscience of meditation interposed by twelve 1-hour each practical sessions of *Integral Meditation*.

The main difference between our intervention approach and classical MPBs is that MBPs (e.g., MBSR and MBCT) have been developed for particular issues such as stress and depression in a clinical setting, while our program was aimed to promote personal and spiritual growth within the general population. Nevertheless, there are many common features between MBPs and our intervention, according to the metaphor of “wrap” and “weft” used to represent the fabric of MBP (Crane et al., 2017), which we discussed in details in Supplementary Material. The *Integral Meditation* used in the current study incorporates different oriental meditation techniques (e.g., Vipassana, Tibetan, and Transcendental, etc.). Its integration of mindfulness with compassion and kindness are manifested primarily in the relationship with oneself, concerning internal peace, genuine well-being and self-esteem mediated by the ability of acceptance and openness to life. Our *Integral Meditation* simultaneously uses breathing, focused attention, release of physical tensions, thoughts, feeling sensations through internal senses and imagery. It eases quick relaxation and physical well-being, and more deeply an energetic and spiritual well-being. The experience has shown that our program is well accepted by both novice and experienced meditators who become familiar with the technique very quickly.

Each meditation session was accompanied by the sound of Tibetan Bowls which invokes a deep state of relaxation, naturally assisting one as he/she enters into meditation. Meditating on the subtle sound of the Tibetan singing bowl helps to tune one into the universal sound within and without.

As for the theoretical part of the intervention, the program comprised five theoretical lessons, each of 4 h, about neuroscience of meditation given by different university professors (see Supplementary Material for further details).

### Self-Report Measures

#### Core Outcome in Routine Evaluation-Outcome Measure

The Core Outcome in Routine Evaluation-Outcome Measure (CORE-OM) (Evans et al., 2000) is a self-report questionnaire with good psychometric properties composed by 34 items aimed to measure the global distress of the subject across three dimensions: *subjective well-being* (4 items), *problems/symptoms* (12 items), *life functioning* (12 items). In addition, there are 6 items on *risk to self and others* that are not regarded as a scale but more as a clinical flag; for this reason we also reported results for *all non-risk items* without this subscale. Item score ranges from 0 to 4. As a clinical tool, the CORE-OM can be used for an initial screening of the patient to track his/her changing over time.

The full-scale mean or the all non-risk items mean among non-clinical sample can be read as a global index of distress while each subscale mean can be used as an index of distress in its specific dimension. A decrease in the mean score after the intervention indicates a diminished global distress or diminished distress relative to the subscale.

The CORE-OM has good psychometric properties and the same applies to the Italian version of the CORE-OM (Palmieri et al., 2009) used in our study.

The original version of the questionnaire reports a mean score of 0.76 (SD = 0.59) among the non-clinical population for all item dimensions (Core System Group, 1998).

### Five-Facet Mindfulness Questionnaire

The Five-Facet Mindfulness Questionnaire (FFMQ) is a 39-item multidimensional assessment tool designed to measure a person’s level of mindfulness (Baer et al., 2006). The subject gives a rating for each item in a five-point Likert scale. The FFMQ is aimed to measure five interrelated components of mindfulness, which are: (1) *observing* (noticing or attending to internal and external experiences), (2) *describing* (labelling internal experiences with words), (3) *acting with awareness* (attending to own activities of the moment and it can be contrasted with behaving mechanically while attention is focused elsewhere), (4) *non-judjing of inner experiences* (taking a non-evaluative stance toward thoughts and feelings), and (5) *non-reactivity to inner experience* (the tendency to allow thoughts and feelings to come and go, without getting caught up in or carried away by them).

The questionnaire has showed good psychometric properties both in the English and Italian version, which also shows a similar factorial structure compared to the original one (Giovannini et al., 2014). The Italian validation study also report that the item mean score in an Italian sample (*N* = 559) is 3.31 (SD = 0,39) (Giovannini et al., 2014). A higher score after an intervention reflects an improved level of mindfulness.

Here we report both the total scores given by the mean scores of all items (i.e., general self-report mindfulness) as well as the mean item-specific domain scores.

### Emotion Regulation Questionnaire

The Emotion Regulation Questionnaire (ERQ) is a 10-item scale designed to measure respondents’ tendency to regulate their emotions in two ways: (1) *cognitive reappraisal* and (2) *expressive suppression* (Gross and John, 2003). Respondents answer each item on a 7-point Likert-type scale ranging from 1 (strongly disagree) to 7 (strongly agree). The items that make up the *cognitive reappraisal* facet are 6, while the items that make up the *expressive suppression* facet are four. The psychometric properties of the questionnaires are good and we used the Italian translation of ERQ (Balzarotti et al., 2010).

The theoretical structure behind this questionnaire is based on evidences that there are different strategies employed for regulate emotional response, and they could work consciously or unconsciously. Despite the existence of many strategies, the ERQ investigates only two of them. *Cognitive reappraisal* is a form of cognitive change which involves construing a potentially emotion-eliciting situation in a way that changes its emotional impact (Lazarus and Alfert, 1964) while *expressive suppression* is a form of response modulation that involves inhibiting ongoing emotion-expressive behavior (Gross, 1998). We analyzed the mean scores of the items-specific domains. A higher score after the intervention in each subscale indicates a more frequently use of that emotional regulation strategy.

The original study (Gross, 1998) reports that the mean score for the *suppression* domain is 3.39 (SD = 1.14) whilst for the *reappraisal* domain is 4.6 (SD = 0.98) on American undergraduates.

### Daily Spirituality Scale

The DSES (Underwood and Teresi, 2002) is a 16-item self-report measure designed to assess ordinary experiences of connection with the transcendent measuring the component of religiousness and spirituality in daily life.

The first 15 items of the questionnaire are measured on a 6-point Likert-type scale; while item 16, which is about feelings of closeness to God, is measured on a 4-point scale.

Items are all coded in the direction that higher score reflects greater level of daily spiritual experiences. For each respondent, scores are summed and then averaged across all the items.

The questionnaire includes constructs such as awareness, gratitude, mercy, sense of connection with the transcendent and compassionate love. It also includes measures of awareness of discernment/inspiration and a sense of deep inner peace.

It was constructed to reflect an overlapping circle model of spirituality/religiousness and contains items that are more specifically theistic in nature, as well as items to tap the spiritual experience of those who are not comfortable with theistic language.

The DSES has good psychometric properties in its original version and here we use the translation to Italian language.

In our study, given our intervention is fairly short (12 sessions), we expected that a dimension such the spirituality could not change since it would take a longer practice of meditation. For this reason, we collected the subject specific score DSES at baseline as a covariate to include in each regression model in order to adjust the estimates of the effect of meditation on each outcome for the baseline level of spirituality.

### Statistical Analysis

Linear mixed models effects (LME) (Pinheiro and Bates, 2000) have been applied to evaluate pre-post treatment changes on each outcome measurement. A subject random effect (1| id) has been used to adjust the models for intra-subject variability produced by the two repeated measurements (at *t*_0_ and *t*_1_) carried out on the same patients (41 patients × 2 measurements = 82 observations but only 41 of them are independents). The model parameters are interpreted as change of the outcome variable over time. *P*-values < 0.05 on a 2-sided test are considered as statistically significant. All models were adjusted for sex, age, previous meditation exprience and the mean score obtained at DSES. Lastly, pairwise partial correlations, adjusted for sex and age (*z*), between the mean score obtained at DSES (*x*) and all psychological questionnaires’ scores obtained at *t*_0_ (*y*), *r*(*x*, *y*| *z*), were calculated, in order to investigate the relationship between the levels of spirituality in our sample and psychological indicators and well-being.

Statistical analysis was performed using R Statistical software (lme4 R package) (R Development Core Team, 2017). Missing data (total % = 0.46) were imputed using ItemMean function of TestDataImputation R package. Questionnaire internal consistency was assessed via Cronbach’s *α* coefficients (Cicchetti, 1994).

## Results

Forty-one subjects were recruited into the study: 17 men (41.46%) and 24 women (58.54%), with a mean age of 41.1 years (SD = 16.14). Out of these, 24 had already experienced meditation practice, but only 12 in an ongoing way. As regards the education, thirty-one (76%) subjects had a degree, two a Ph.D. title, while eight had high school diploma only (see Table 1).

**TABLE 1:** Baseline characteristics of the sample.

Variable	*N* (%)
**Age**	
20–30	17 (41%)
31–50	10 (24%)
>51	14 (34%)
**Sex**	
Male	17 (41%)
Female	24 (59%)
**Previous meditation experience**	
Yes	24 (59%)
No	17 (41%)
**Type of meditation (n = 24)**	
Mindfulness	5 (12%)
Gassho	1 (4%)
Transcendental	5 (12%)
Vipassana	2 (8%)
Yoga	7 (17%)
Zen Zazen	2 (8%)
NA	2 (8%)
**Education**	
High school	8 (19%)
Degree	31 (76%)
Ph.D.	2 (5%)

For each subject, questionnaires were handed out both *t*_0_ and *t*_1_, except DSES that was measured only at *t*_0_. In our sample, the mean (±SD) of DSES was 3.02 (±0.97), and the internal consistency was excellent (α = 0.96). Based on the 3rd quartile of DSES, nine subjects (6 females and 3 males) showed a high spirituality (>3.69), while 32 subjects (18 females and 14 males) showed a low spirituality (≤3.69).

As regard to the CORE-OM questionnaire, its internal consistency was good both at *t*_0_ and at *t*_1_ for the following domains: *subjective well-being* (*t*_0_ α = 0.68, *t*_1_ α = 0.81), *life functioning* (*t*_0_ α = 0.82, *t*_1_ α = 0.85), and *problems/symptoms* (*t*_0_ α = 0.85, *t*_1_ α = 0.91). With regards to *risk to self and others* dimension the Cronbach’s *α* coefficients, in our sample, did not reach an acceptable value (*t*_0_ α = 0.48, *t*_1_ α = 0.88). The items included in the risk domain are considered clinical flag which requires attention in a clinical evaluation. In the current study we did not use the CORE-OM as an assessment tool, so we decided to report both the *all items* and *all non-risk items* results as suggested in the CORE-OM user’s manual. The mean score of CORE-OM is perfectly in line with the interval of the general non-distressed population (0.0–1.9) (Connell et al., 2007).

As regard to the FFMQ questionnaire, its internal consistency was good both at *t*_0_ and *t*_1_ for all five domains: *observing* (*t*_0_ α = 0.86, *t*_1_ α = 0.87), *describing* (*t*_0_ α = 0.92, *t*_1_ α = 0.94), *acting with awareness* (*t*_0_ α = 0.89, *t*_1_ α = 0.93), *non-judging of inner experiences* (*t*_0_ α = 0.84, *t*_1_ α = 0.93), and *non-reactivity to inner experience* (*t*_0_ α = 0.83, *t*_1_ α = 0.85).

As regard to the ERQ questionnaire, its internal consistency was good both for *cognitive reappraisal* at *t*_0_ (α = 0.82) and *t*_1_ (α = 0.87) and for *expressive suppression* at *t*_0_ (α = 0.71) and *t*_1_ (α = 0.82), showing that reliability of the instrument is stable during the whole survey. Descriptive statistics of the questionnaires scores at *t*_0_ and *t*_1_ are reported in Table 2; while the results of the LME model for the general outcome and the different domains are reported in Table 3.

**TABLE 2:** Summary of the pre/post intervention outcome measurement: mean (SD), median (min, max), and internal consistency.

		Pre-intervention (*t*_0_)			Post-intervention (*t*_1_)		
		
Questionnaire	*n*.	Mean (SD)	Median (min–max)	Internal consistency	Mean (SD)	Median (min–max)	Internal consistency
**CORE-OM**							
All Items	41	0.98 (0.48)	0.91 (0.32–2.5)	0.92	0.82 (0.55)	0.65 (0.09–2.41)	0.94
AlI non-risk items*	41	1.18 (0.57)	1.11 (0.39–3.03)	0.92	0.98 (0.63)	0.79 (0.11–2.46)	0.94
Subjective well-being	41	1.28 (0.72)	1.25 (0–3)	0.68	1.05 (0.82)	0.75 (0–3.25)	0.81
Life functioning	41	1.09 (0.56)	0.92 (0.42–2.83)	0.82	0.92 (0.55)	0.83 (0–2.33)	0.85
Problems/Symptoms	41	1.24 (0.63)	1.25 (0.25–3.25)	0.85	1.01 (0.76)	0.92 (0–2.75)	0.91
Risk to self and others	41	0.07 (0.14)	0 (0–0.5)	0.48	0.11 (0.39)	0 (0–2.17)	0.88
**FFMQ**							
All items	41	3.54 (0.49)	3.54 (2.49–4.79)	0.93	3.70 (0.56)	3.72 (2.51–4.82)	0.95
Observing	41	3.78 (0.65)	3.87 (2.12–5)	0.86	3.81 (0.73)	3.87 (2–4.87)	0.87
Describing	41	3.80 (0.70)	3.87 (2.37–5)	0.92	3.91 (0.80)	4.12 (2.37–5)	0.94
Acting with awareness	41	3.39 (0.71)	3.29 (2–4.71)	0.89	3.61 (0.84)	3.71 (2–5)	0.93
Non-reactivity to inner experience	41	3.20 (0.58)	3 (2.14 – 4.71)	0.83	3.20 (0.70)	3.14 (2–4.86)	0.93
Non-judging of inner experiences	41	3.53 (0.69)	3.62 (2.37 – 5)	0.84	3.91 (0.80)	4 (2.25–5)	0.85
**ERQ**							
Expressive suppression	41	3.30 (1.12)	3.25 (1–5.50)	0.71	3.06 (1.30)	3 (1–5.75)	0.82
Cognitive reapprasal	41	4.89 (0.98)	5 (1.67–6.33)	0.82	5.43 (0.99)	5.50 (3.17–7)	0.87

SD, Standard deviation; CORE-OM, Core Outcomes in Routine Evaluation-Outcome Measure; FFMQ, Five Facet Mindfulness Questionnaire; ERQ, Emotion Regulation Questionnaire.

*excluding *risk to self and others* subscale.

*Post intervention higher CORE-OM score indicates deteriorating mental health and more distress. Increased post intervention FFMQ scores reflect increased levels of mindfulness.*

**TABLE 3:** Linear mixed model: for each questionnaire β coefficient with its 95%CI, standard error, and *P*-value for the null hypothesis of β = 0 are reported.

Questionnaire	β [95%CI]	Standard error	*P*-value
**CORE-OM**			
All Items	−0.159 [−0.34;0.02]	0.488	0.087
AlI non-risk items*	−0.202 [−0.41;0.008]	0.104	0.059
Subjective wel-lbeing	−0.226 [−0.50;0.05]	0.137	0.108
Life functioning	−0.171 [−0.35;0.005]	0.087	0.057
Problems/Symptoms	−0.228 [−0.48;0.024]	0.125	0.075
Risk to self and others	0.044 [−0.07;0.16]	0.060	0.462
**FFMQ**			
All item	0.154 [0.04;0.27]	0.058	0.012
Observing	0.027 [−0.14;0.19]	0.081	0.737
Describing	0.113 [−0.005;0.23]	0.058	0.061
Acting with awareness	0.212 [0.03;0.40]	0.091	0.024
Non-reactivity to inner experience	−0.003 [−0.22;0.21]	0.106	0.974
Non-judging of inner experience	0.384 [0.21;0.56]	0.088	0.0001
**ERQ**			
Expression suppression	−0.238[−0.56;0.09]	0.16	0.145
Cognitive reappraisal	0.541[0.26;0.82]	0.137	0.0003

P-values < 0.05 are considered as statistically significant. All models are adjusted for sex, age, spirituality, and previous meditation experience. All the covariates were not significant, and their effects were not reported. *Excluding risk to self and others subscale.

No statistically significant pre-post change at the 5% conventional level were observed for the CORE-OM, although the negative sign of the β coefficients reveals that the direction is towards a beneficial effect; while statistically significant results were obtained for *all items* of FFMQ (β = 0.154, *P*-value = 0.012), *acting with awareness* (β = 0.212, *P*-value = 0.024) and *non-judging of inner experiences* (β = 0.384, *P*-value = 0.0001) subscales and for *cognitive reappraisal* subscale of ERQ questionnaire (β = 0.541, *P*-value = 0.0003).

Lastly, we have assessed the pairwise partial correlations, adjusted for age and sex, between DSES mean score and the psychological indicators’ scores measured at *t*_0_ (Table 4). A significant correlation has been found between DSES score and *life functioning* CORE-OM domain at pre-intervention (*r* = −0.48, *p* = 0.002).

**TABLE 4:** Partial correlation *r*(*x*, *y*| *z*) between DSES (*x*) and the indicators under study (*y*) at pre-intervention (*t*_0_) adjusted for sex and age (*z*).

Variables	*r*(*x*, *y*| *z*)	95% CI	*P*-value
**CORE-OM**			
All Items	–0.12	−0.42; 0.20	0.46
AlI non-risk items	–0.12	−0.42; 0.21	0.48
Subjective well-being	–0.25	−0.52; 0.07	0.12
***Functioning***	−**0.48**	−0.69; −0.19	**0.002**
Problems/Symptoms	–0.28	−0.55; 0.04	0.09
Risk to self and others	–0.22	−0.50; 0.10	0.17
**FFMQ**			
All item	0.22	−0.11; 0.48	0.19
Observing	0.29	−0.03; 0.56	0.07
Describing	0.30	−0.01; 0.56	0.06
Acting with awareness	0.28	−0.03; 0.55	0.08
Non-reactivity to inner experience	0.19	−0.13; 0.48	0.25
Non-judging of inner experience	0.26	−0.06; 0.53	0.40
**ERQ**			
Cognitive reappraisal	0.06	−0.25; 0.37	0.06
Expressive suppression	–0.25	−0.52; 0.07	0.13

*P-values < 0.05 are considered as statistically significant.*

## Discussion

The practice of meditation has a very long history and its beneficial effects have been repeatedly observed and reported in the passage of centuries. In the last decades the scientific community has also acquired interest to analyze its effects on human mental and physical health also in non-clinical population.

Some studies have been conducted in Italy among general non-clinical population in order to investigate the effects of mindfulness meditation training on psychosocial well-being and quality of life. Receiving an 8-weeks mindfulness-oriented meditation training leads to different outcomes in terms of mindfulness skills, personality traits, and religious/spiritual self-representations (Matiz et al., 2018). A study by Campanella et al. (2014) brought evidences that an 8-week mindfulness-mediation program has a positive effect on the personality profiles: they found that their training was sufficient to modify the character description the people give of themselves especially related to the dimensions of self-concept. Another study (Romano et al., 2014) conducted on psychology students showed that a 6-weeks training of mindfulness produced an increased level of mindfulness, and an improving in the ability of decentering, that includes the one’s ability to look at his own thoughts and feelings as transient mental events that do not necessarily reflect the reality.

In our study 41 subjects recruited from the general population underwent a short-term intervention which includes *Integral Meditation* and lectures on neuroscience of meditation. Our work aimed at evaluating the within-subject (pre vs post) effect of the intervention on psychological indicators, measured using the standardized Italian version of three selected self-report questionnaires (i.e., CORE-OM, FFMQ, and ERQ), handed out before the start of and after the last session of the intervention.

The self-report questionnaires are handy instruments and provide valuable evaluative information about the individual, based on a person’s report without any interference from the researcher. The use of these questionnaires is a significant advance in the study of meditation as the process of writing items for any self-report questionnaire requires authors to define or conceptualize the construct they are attempting to measure (Baer et al., 2006).

As to the CORE-OM questionnaire, the way it is designed indicates that the higher the score, the more troubled the patient is in terms of global distress, and vice versa. In our study, the descriptive statistics calculated in the sample showed a declining pattern from pre- to post-intervention in *life functioning*, *subjective well-being*, and *problems/symptoms* domains and a general decline if we consider all the items together. In terms of mean and median pre- vs post- scores, the most important decline, which translates to evidence of major beneficial effects, was observed in *subjective well-being* domain. The CORE-OM *risk to self and others* domain showed, instead, a low value of Cronbach’s *α* coefficients but, given the study was performed on a healthy population, the results for this subscale are not very relevant. The results obtained for all *non-risk items* indicate a general improvement in the global distress even if, probably due to the small sample size, the calculated *P*-value (*P* = 0.06), although very close to the significant threshold, did not reach the chosen nominal statistical significance (*P* < 0.05).

Regarding the FFMQ questionnaire, an increase in the scores subsequent to the intervention reflects an increased level of mindfulness. Also for this outcome, the descriptive statistics showed an increasing trend in the scores from pre- to post- intervention, both considering all items together and the different domains separately. This trend was statistically significant for *non-judging of inner experiences* (β = 0.984, *p* = 0.0001) and *acting with awareness* (β = 0.212, *p* = 0.02) domains, and for all items taken together (β = 0.154, *p* = 0.012), thus showing a clear positive effect of the intervention.

Lastly, we examined the ERQ questionnaire, evaluating two emotion regulation strategies: *cognitive reappraisal* and *expressive suppression*. Emotions regulation is of great importance when analyzing different aspects of healthy adaptation from affective functioning to social relations. In general, habitual *expressive suppression* is associated with an increase in the incidence of mental health problems. Thus, observing a lower degree of habitual *expressive suppression* and a greater degree of habitual *cognitive reappraisal* is linked to decreased negative emotions and increased positive emotions. In our sample we observed a decreased mean score from pre- to post-treatment in the *expressive suppression* domain even if not statistically significant (*p* = 0.145), the change as measured by the 95% CI of the β coefficient is shifted towards negative values; while *cognitive reappraisal* domain showed an important increase in the post-treatment score’s mean and median as compared to pre-treatment, reaching statistical significance (β = 0.54, *p* = 0.0003), thus suggesting a role of the intervention in acting on increased reappraisal domain.

It is important to notice that the self-awareness and emotions regulation are strictly related to each other (Silvia, 2002). Indeed, self-awareness is considered to be a pre-condition for the use of reappraisal as an adaptive emotion regulation strategy (Subic-Wrana et al., 2014). In line with previous neuroimaging evidence, one might also speculate that a short-term meditation training could modulate these two domains acting on specific cortical-subcortical neural substrate, involving the prefrontal cortex and amygdala (Herwig et al., 2010). Nevertheless, specific neuroimaging studies are needed to better explore the neural substrate of such behavioral change.

Many published studies (Keefe et al., 2001; Fowler and Hill, 2004; Holland and Neimeyer, 2005; Bonelli et al., 2012) have investigated the connection between spiritual experiences, as measured by DSES, and multiple dimensions of psychological well-being (e.g., distress, anxiety, depression), health outcomes (e.g., substance abuse, arthritis rheumatoid), and life satisfaction. DSES positively influence cancer survivors’ well-being and lifestyle (Park et al., 2009), helping controlling pain during cancer treatment (Lo et al., 2016) providing a source of strength and comfort. In general, spiritual well-being is considered a determinant factor influencing individuals’ quality of life (Holland and Neimeyer, 2005). Investigating the correlation between baseline DSES and the psychological indicators’ scores measured at *t*_0_ allowed to identify in our sample a statistically significant negative correlation between DSES and *functioning* CORE-OM domain at pre-intervention (*r* = −0.48, *p* = 0.002), this result is line with literature (Paine et al., 2018) and indicates the importance of including DSES as a covariate in the model.

Application of our intervention could be particularly efficient among those people who are exposed to work-related stress, leading to impairment such as cognitive decline (Giorgi et al., 2020). Those detrimental effects of stress are reflected both on work performance and quality of life, with higher risk for elder people who are more likely to develop cognitive impairment. Meditation practice has been shown to be an effective exercise in preventing the age-related cognitive decline (Sperduti et al., 2017) and the risk of developing cognitive impairment such as Alzheimer Disease (Khalsa, 2015). Another remarkable application of our intervention could be among healthcare professionals. The work conditions of the latter are often not adequate (Kowalczuk et al., 2019) or high requiring of physical and mental resources, with a negative impact on their performances and health (Raab, 2014). Meditation training has been demonstrated to be an economic and efficient way to improve well-being and to reduce burnout in healthcare professionals, in improving their professional skills as well as to increase patient outcomes (Raab, 2014; Rao and Kemper, 2017).

Given the benefits that meditation brings we can argue that MBI should be considered by managers and organizations as a mean to improve the health, quality of life and performance of the employees, in particular to those exposed to job stress or cognitive decline. Being our *Integral Meditation* program based on an easy-to-learn technique that brings quick benefits to the practitioners, it can be attractive and motivational to naive meditators or to those who are not motivated to meditate; moreover, the theoretical lectures included in our intervention can be helpful to generate and maintain motivation of those who are skeptic or not particularly interested in meditation.

In accordance with our a-priori hypothesis, in this study we obtained evidence that practicing meditation in a big group and jointly learning the theoretical and scientific aspects of meditation may have a very positive effects such as improve different aspects of psychological functioning and reduce stress that is thought to be at the basis of numerous medical conditions; anyway our findings must be considered in light of several limitations.

Beside the behavioral measures of our study are linked to the psychological construct of well-being, we didn’t directly measured participants’ well-being. In this sense, we have information about the effects that our intervention has on specific domains of psychological functioning, but we do not have information regarding the impact of those effects on well-being and so we cannot directly state that those changes have effectively led to an improvement in the participants’ quality of life. Future investigations may help to clarify if our intervention leds to improvements in well-being and quality of life directly measured with appropriate tools. Moreover, being our intervention composed by an original meditation program which includes aspects from different meditation techniques and particular features such as Tibetan Bowls it might be more difficult to reproduce in other studies compared to the classical and standardized MBI interventions such as MBSR program.

Our study was based on a one-group pretest-posttest research design. The choice of this kind of study was driven by the small sample size availability and by the practical necessities imposed by limited funding. The limitations are mainly related to the study design: the lack of a control group, the small sample size and the presence of threats to internal validity (e.g., regression to the mean). These limitations do not allow us to draw causal conclusions on the effect of the intervention on the outcomes analyzed, as confounding factors could have influenced the outcomes other than the investigated intervention (Shadish et al., 2002). A major limitation of small sample studies is that they can produce false-positive results, or they can over-estimate the magnitude of an association, thus they do not normally yield reliable or precise estimates. Anyway results derived from an hypothesis-generating study and so data from our study can be used to design a larger confirmatory study (Hackshaw, 2008). Moreover, in our study the interval between pre- and post- measurements was quite short, as this is a short-term intervention, and this could improve the internal validity, limiting the within-subject variation due to chance. Another limitation is given by the sole use of self-report questionnaires (“soft” measures), which can give inflated results due to the fact that they can potentially be answered in a positive manner even after the first taste of meditation as they strongly correlate to the subject’s mood and expectation demand. The opposite is also true in the case of participants who are extremely sceptical about the method (Goleman, 2018). Such biases are less probable to affect physiological processes, not measured here, like heart rate or brain activity, which are considered hard metrics.

Furthermore, we should be caution when generalizing the results since our sample was recruited among people who were probably interested and motivated toward theoretical and practical aspects of meditation before starting the intervention. Motivation plays a key role in the success of such intervention since meditation requires high individual work: subjects recruited from the general population that don’t share with our sample the same interest toward meditation may not benefit from the intervention in the same way. So, our results can be only generalized to individuals from the general non-clinical population who are motivated to meditate. Anyway, including in our intervention theoretical and reliable scientific information about meditation may lead to an increased motivation even among those who don’t are motivated per se.

Despite the limitations discussed above, this study offers the opportunity to compare the difference between pre- and posttreatment measurements and our results provide evidence of beneficial effects of our short-term intervention (*Integral Meditation* and theoretical lectures) on psychological indicators in subjects from general population and encourage its practice in a big group.

## Conclusion

This study represents a pilot study that aimed to test the effectiveness of an original MBI in improving psychological functioning of an adult non-clinical population, and few studies of this kind have been conducted among Italian population. The results are in accordance with our a-priori hypothesis regarding the beneficial effects of a short-term meditation training and constitute further evidence of the usefulness of this kind of intervention among non-clinical populations. Furthermore, the results provide preliminary support for the efficacy of our intervention, that can be used to design a larger confirmatory study.

## Data Availability Statement

All datasets presented in this study are included in the article/Supplementary Material.

## Ethics Statement

All procedures performed in studies involving human participants were in accordance with the ethical standards of the institutional and/or national research committee and with the 1964 Helsinki declaration and its later amendments or comparable ethical standards. This manuscript does not contain any studies with animals performed by any of the authors. Informed consent was obtained from all individual participants included in the study.

## Author Contributions

LB designed the study. TF performed the statistical analysis. SB conducted the meditation session. FB, GB, GS, II, and GB critically interpreted the results. GB revised the final version of the manuscript as to the English language. All authors contributed to writing of the manuscript and have approved the final manuscript.

## Conflict of Interest

The authors declare that the research was conducted in the absence of any commercial or financial relationships that could be construed as a potential conflict of interest.
