# Supplementary material for: Short-Term Meditation Training Fosters Mindfulness and Emotion Regulation: A Pilot Study
Source: Front Psychol. 2020 Oct 26;11:558803. doi: 10.3389/fpsyg.2020.558803 (PMC7649763; doi:10.3389/fpsyg.2020.558803)
Supplement: Supplementary file 2 [file Data_Sheet_2.docx]

Supplementary Material

**Intervention**

Our program, named *Integral Meditation*, is spiritual in nature and shares the following “*wrap*” features with MBP:

**a)** it uses mindfulness practices as a vehicle for a systematic training of the mind in the service of developing greater awareness of self and others and it is informed by theories and practice that draw from contemplative traditions, while leaving behind their religious, esoteric and mystical elements;

**b)** it is underpinned by a model of human experience which addresses that causes of human distress and the pathways to relieve it;

**c)** it develops a new relationship with experience characterized by focusing on present moment, and on decentring (i.e. by considering thoughts and feelings as mental events which come and go in the mind as clouds in the sky). The training enables the participants to make a radical shift to their thoughts, feelings and body sensations, as well as to outer circumstances;

**d)** supports the development of greater attentional, emotional and behavioural self-regulation, as well as positive qualities such as compassion, wisdom and equanimity by cultivating an internal climate of friendliness towards experience whether it be pleasant or unpleasant;

**e)** the training develops familiarity with and understanding of the mind and body and appreciation that attention can be regulated, fine-tuned, and optimized through training. Rather like physical training, the training progresses developmentally and sequentially throughout the program.

The “*weft*” feature of our program resides in:

1. using the imagery to power the concentration and to change the brain waves from beta to alpha/theta leading to a different state of consciousness;
2. using of Tibetan Bowls which invoke a deep state of relaxation which naturally assists one in entering into meditation. They are a quintessential aim to meditation and can be found on private altars, and in temples, monasteries and meditation halls through the world. Meditating on the subtle sound of the Tibetan singing bowl tunes one into the universal sound within and without. Each meditation session from phase 1 to phase 4 was accompanied by this sound.
3. being scheduled over 12 weekly guided sessions lasting 60 minutes each. The 12 sessions are structured into 4 cycles, each cycle comprises three subsequent meditation sessions. Each cycle is focused on a specific ability as reported below:

Cycle 1: aware diaphragmatic breathing, keeping the posture.

Cycle 2: body scan and awareness of body sensations

Cycle 3: emotions and thoughts feeling and releasing

Cycle 4: imagery activity to change the state of consciousness.

Each cycle also comprises the abilities acquired in the preceding cycle/s, as a result there is an evolution across cycles.

The last cycle, i.e. Cycle 4, also comprises all the abilities acquired in the preceding cycles and is hence structured into the following six final phases:

1. keeping **posture** during meditation, focusing on an aware **diaphragmatic breathing,** body **scan** and awareness of sensations to induce body relaxation leading to an inner emotional calm that is tightly linked to the calm of the physical body; (abilities acquired in Cycle 1 and Cycle 2) *Duration 10 minutes*
2. **feeling and examination of emotion and thoughts**; (abilities acquired in Cycle 3) *Duration 5 minutes*
3. **visualization of images** (e.g., a sphere of light), colours, relaxing landscapes (e.g., a garden). Visualization powers concentration and calms negative and disturbing dominant emotions. This is not yet a meditation state, but a state leading to the true meditation experience; (abilities acquired in Cycle 4) *Duration 20 minutes*
4. **a time of silence** to enjoy the new state of consciousness. After phase iv) the person is free of body sensations, emotions and mental processes and ready to expand his/her own consciousness and open fully him/herself with trust to the union with the higher self and the unified universal field, thus entering into a fine state of meditation to enjoy his/her own personal experience; (abilities acquired in Cycle 4) *Duration 15 minutes*
5. **reconnection to the feeling** of the body sensations; (abilities acquired in Cycle 4) *Duration 5 minutes*
6. **awareness of the new mental and physical** state (well-being, happiness, peacefulness, serenity etc.). (abilities acquired in Cycle 4) *Duration 5 minutes*
7. **sharing the experience:** at the end of each meditation class, the trainer asks the participants how they feel to allow them to freely share their feelings and impressions about the meditation experience.
8. **practice at home:** participants were highly encouraged to practice on their own guided by audio-files on recorded meditation lasting 30 minutes each.

As for the theoretical part of the intervention, the program comprised fifth frontal lessons about neuroscience of meditation given by different university professors.

The *first* lesson was a general introduction to the meditation, from religion, literature and art to the science and neuroscience point of view. The *second* lesson covered the topic of emotions and brain (what they are, how they work) and the different strategies that humans use to regulate emotions, included mindfulness. The *third* lesson was about the stress circuit and how mindfulness can influence stress, presenting the MBSR. The *fourth* lesson explored the biological correlates of meditation (neurotransmitters, hormones, neurotrophic factors) and their role in providing scientific evidences that support different types of meditation. The *fifth* lesson introduce the statistical methods in the research of meditation.
